# Brief Story on Prostaglandins, Inhibitors of their Synthesis, Hematopoiesis, and Acute Radiation Syndrome

**DOI:** 10.3390/molecules24224019

**Published:** 2019-11-06

**Authors:** Michal Hofer, Zuzana Hoferová, Martin Falk

**Affiliations:** Department of Cell Biology and Radiobiology, Institute of Biophysics, v.v.i., Czech Academy of Sciences, Královopolská 135, 61265 Brno, Czech Republic; hoferovaz@centrum.cz (Z.H.); falk@ibp.cz (M.F.)

**Keywords:** prostaglandins, inhibitors of prostaglandin synthesis, cyclooxygenase, acute radiation syndrome, hematopoiesis, gastrointestinal system

## Abstract

Prostaglandins and inhibitors of their synthesis (cyclooxygenase (COX) inhibitors, non-steroidal anti-inflammatory drugs) were shown to play a significant role in the regulation of hematopoiesis. Partly due to their hematopoiesis-modulating effects, both prostaglandins and COX inhibitors were reported to act positively in radiation-exposed mammalian organisms at various pre- and post-irradiation therapeutical settings. Experimental efforts were targeted at finding pharmacological procedures leading to optimization of therapeutical outcomes by minimizing undesirable side effects of the treatments. Progress in these efforts was obtained after discovery of selective inhibitors of inducible selective cyclooxygenase-2 (COX-2) inhibitors. Recent studies have been able to suggest the possibility to find combined therapeutical approaches utilizing joint administration of prostaglandins and inhibitors of their synthesis at optimized timing and dosing of the drugs which could be incorporated into the therapy of patients with acute radiation syndrome.

## 1. Introduction

Prostaglandins, as well as inhibitors of their synthesis, act in hematopoiesis through a spectrum of pleiotropic effects [1,2]. Therefore, there is no wonder that both groups of substances have appeared among those tested as potential modulators of radiation damage in mammals [3]. One of the proposed needs for the development of efficient and non-toxic modulators of radiation damage is that of the necessity to apply such drugs in connection with contingent radiation accidents or terrorist attacks [4,5,6].

The story indicated in the title began in the 1970s and 1980s. At that time, reports appeared informing nearly simultaneously about protective effects of either prostaglandins or inhibitors of their synthesis on ionizing radiation-induced acute radiation damage of the mammalian organism. The same period is characterized also by emerging studies on hematological effects of both the groups of substances mentioned [7,8].

Subsequent studies, especially of radiobiological and hematological targeting, brought new pieces of information about the contribution of prostaglandins and inhibitors of their synthesis to the regulation of hematopoiesis under normal and perturbed conditions. Some of these studies also uncovered new possibilities on how to enhance recovery following an exposure to sublethal or lethal radiation doses [9,10]. Significant progress in the methodological spectrum of the studies on the above topics was obtained when selective inhibitors of cyclooxygenase-2 (COX-2), one of the enzymes of the prostaglandin synthesis pathway, appeared [2].

This article deals with the outlined story and shows where the story can be found at present.

## 2. Hematological Effects of Prostaglandin E_2_ (PGE_2_)

Prostaglandin E_2_ (PGE_2_) was shown to dose-dependently inhibit mouse and human myeloid progenitor cell proliferation in semisolid culture assays [11,12]. The same finding was obtained during in vivo studies and a hypothesis was postulated that prostaglandins played an important role in the negative hematopoietic feedback control [2,13,14,15,16]. In a later study, the inhibitory effect of PGE_2_ on granulocyte–macrophage progenitor cells was shown to synergize with that of interferons and to be mediated by tumor necrosis factor [17].

In contrast to the inhibitory action of PGE_2_ on myeloid progenitor cells, this substance was repeatedly reported to stimulate proliferation of the erythroid progenitor cells [18,19,20,21,22]. The effect of PGE_2_ could be direct or mediated through factors released by T cells [23].

In vitro studies from 1982 have reported a stimulated production of cycling hematopoietic progenitor cells from a population of quiescent, non-cycling cells, most likely of stem cells from mouse or human bone marrow exposed to PGE_2_ [24]. Later findings from 1998 have shown an increased formation of both myeloid and erythroid progenitor cells from purified human blood CD34^+^ cells [25]. PGE_2_ has been reported to increase hematopoietic stem cell long-term-engraftment [26] and homing efficiency [27], to decrease their apoptosis [26,27], as well as to increase their entry into the cell cycle [26].

As shown above, PGE_2_ influences various components of the hematopoietic system in different directions. Therefore, the resulting hematological effects of PGE_2_ are manifestations of complex regulatory PGE_2_ actions. Consequently, hematological effects of PGE_2_ cannot be evaluated on a universal “good or bad” or “stimulatory or inhibitory” basis, but only from partial viewpoints and taking into account dosing and timing of the drug, evaluation time interval, the way of prostaglandin action (in vitro, ex vivo, in vivo) etc. Detailed analysis of methodological aspects of hematological studies on PGE_2_ and other eicosanoids, as well as of mechanisms of their effects, can be found in a separate review [1].

## 3. Concise Overview of Acute Radiation Syndrome

Acute radiation syndrome (radiation sickness) is caused by exposure of a mammalian organism to a high dose of penetrating, ionizing radiation over a short period of time [28]. Differences in cellular sensitivity to ionizing radiation underlie the classic division of the acute radiation syndrome into three syndromes characterized by the extent of radiation dose by which they are produced, namely hematopoietic (bone marrow) syndrome, gastrointestinal syndrome, and neurovascular syndrome [28]. In man, the hematopoietic radiation syndrome is caused by radiation doses between 2 and 6 Gy [28]. This syndrome is the most probable to appear, e.g., as a consequence of radiation accidents. When appropriately treated by hematological interventions, survival is possible [29]. If untreated, about half of all people exposed to a dose of more than 3.5 Gy will die within 60 days from infection and bleeding [30]. From the clinical importance of the hematopoietic radiation syndrome, the emphasis on hematological pharmacological intervention in this article can be deduced. The gastrointestinal syndrome is diagnosed between radiation doses of 6 to 10 Gy and survival is possible in the lower part of this dose range [29], but most of the patients succumb after irradiation with doses in this range within weeks of exposure [31]. The neurovascular syndrome (over 10 Gy in man) is absolutely lethal [28]; at doses in excess of 50 Gy victims will die within 48 h [32].

Radiosensitivity differs between various species [33]. Table 1 shows approximate values of LD_50/30_ (radiation dose that kills 50 per cent of irradiated individuals within 30 days after exposure) for X-ray whole-body irradiation of various mammalian species, including man (for man, the LD_50/30_ cannot be experimentally obtained and, therefore, the value shown was estimated [34]).

As follows from many data, in mouse, which is the experimental species most often used for radiobiological studies, the absolute radiation doses for the individual radiation syndromes are much higher in comparison with man [33].

## 4. Prostaglandins Act Radioprotectively

Much work on the topic of pharmacological modulation of radiation damage by prostaglandins was done by the group of Hanson et al. They showed that various prostaglandin derivatives, like 16,16-dimethyl PGE_2_ [9], or misoprostol, a prostaglandin E_1_ analog [35], protected intestinal stem cells from deleterious effects of ionizing radiation. Of interest was also their finding that the radioprotection by 16,16-dimethyl PGE_2_ was induced not only in the compartment of intestinal stem cells but also in that of the hematopoietic stem cells [36]. This finding is in agreement with those of hematologists [24,25].

A significantly increased survival in mice administered a pre-irradiation dose of 16,16-dimethyl PGE_2_ was also reported [37]; the authors stated that the administration of the drug extended the LD_50/30_ (the radiation dose killing 50 percent of the animals by day 30 after irradiation) from 9.39 Gy in the controls to 16.14 Gy in the mice treated with 16,16-dimethyl PGE_2_ [37]. Further research revealed that misoprostol (a normal tissue protector [35]) did not protect tumors from radiation injury and could, thus, achieve therapeutic gain [38]. Of interest can be the finding of Wang et al. [39] who found that total-body irradiation in the dose range for the hematopoietic radiation syndrome also induced an intestinal injury. Therefore, the radioprotective efficacy of prostaglandins on gastrointestinal tissues can also be beneficial following the radiation exposure within the range of that for the hematopoietic radiation syndrome.

## 5. Cyclooxygenases Carry out Prostaglandin Synthesis, Their Inhibition Can Be Selective

**Figure 1:**
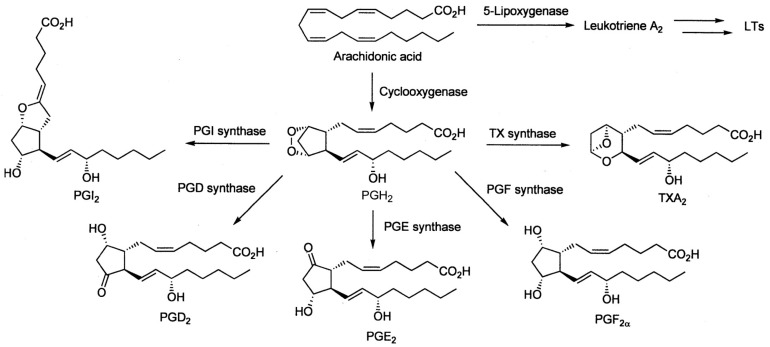
Scheme of prostaglandin and related prostanoid synthesis from arachidonic acid; LTs—leukotrienes; PGI—prostaglandin I; TC—thromboxane; PGD—prostaglandin D; PGH—prostaglandin H; PGE—prostaglandin E; PGF—prostaglandin F; copied from [50].

The formation of prostaglandins from arachidonic acid takes place in a series of steps; prostaglandin H synthase catalyzes the reduction of prostaglandin G_2_ to prostaglandin H_2_ [40]. Prostaglandin H synthase exists in two isoforms, namely cyclooxygenase-1 (COX-1), which is expressed constitutively in a variety of tissues including the gastrointestinal tract, and cyclooxygenase-2 (COX-2), which is inducible and responsible for the production of prostaglandins during inflammatory states [41,42,43,44]. Figure 1 summarizes the synthesis of prostaglandins and related prostanoids from arachidonic acid. Non-steroidal anti-inflammatory drugs (NSAIDs) inhibit the production of prostaglandins by blocking the access of arachidonic acid to the active site of the cyclooxygenases (COXs) [45]. Individual NSAIDs differ in their selectivity for COX-1 and COX-2 but this selectivity is never absolute for one of the COX isoforms [46]; e.g., meloxicam, whose effects will be discussed in detail later, has a six-fold selectivity for COX-2 [46] and it is classified among “COX-2-selective NSAIDs”, but sometimes among “COX-2-preferential NSAIDs” [47]. Experimental and clinical data demonstrated a reduced risk of the undesirable gastrointestinal side effects after administration of COX-2-selective inhibitors as compared with the effects induced by classical non-selective cyclooxygenase inhibitors, as an example, see Reference [48]. The importance of COX-1 for the gastrointestinal tissues was emphasized by Cohn et al. [49] who reported that PGE_2_ produced through COX-1 promoted crypt stem cell post-irradiation survival and proliferation. The selectivity of some of the COX inhibitors can be utilized in modulation of radiation damage in a mammalian organism, as discussed below.

## 6. Effects of Non-Selective COX Inhibitors in Sublethally and Lethally Irradiated Experimental Animals

Since the hematopoiesis-modulating effects of non-selective NSAIDs and the actions of these drugs in the radiation-damaged mammalian organism are discussed jointly in many studies, they will also be dealt with jointly here.

In 1982, indomethacin, a non-selective COX inhibitor, was reported to increase numbers of myeloid progenitor bone marrow cells [51]. Subsequent studies showed that prostaglandins [52] and non-selective NSAIDs [53,54] oppositely influenced production of cytokines by monocytes. Based on these findings it could be suggested that NSAIDs removed the prostaglandin-mediated negative feedback control (see Section 2) of some important hematopoietic compartments.

A number of studies were performed testing the effects of non-selective COX inhibitors on hematopoiesis suppressed by ionizing radiation. The results of these studies are presented in more detail in earlier reviews [2,55]. Briefly, non-selective COX inhibitors, like indomethacin, diclofenac, and flurbiprofen, were reported to enhance mouse hematopoiesis when administered singly before or after one-time-sublethal irradiation, in the course of fractionated irradiation [56,57,58], as well as when given concomitantly with immunomodulators [59,60,61] or chemical radioprotectors [62].

The desirable action of non-selective COX inhibitors did not result in an enhanced survival of experimental mice after their lethal radiation exposure. Reduced survival was found in lethally irradiated mice treated with non-selective COX inhibitors either before [63] or after irradiation [64]. Since non-selective COX inhibitors are known for inducing a high incidence and intensity of undesirable side effects on the gastrointestinal tissues [65,66] and since lethal radiation doses can induce, besides the bone marrow radiation syndrome, also the gastrointestinal radiation syndrome (see Section 3), it could be deduced that it was just the manifestations of the gastrointestinal radiation syndrome which were aggravated by non-selective COX inhibitors in lethally irradiated mice. Thus, possible use of these drugs in the treatment of the acute radiation syndrome in man has been found to be significantly restricted.

## 7. Effects of Selective COX-2 Inhibitors in Sublethally and Lethally Irradiated Experimental Animals

The first report on hematopoiesis-stimulating action of selective COX-2 inhibitors appeared in 1998 when the selective COX-2 inhibitor NS-398 was reported to increase numbers of total white blood cells and neutrophils in experimentally burned rats [67]. Further studies on COX-2-selective inhibitors of prostaglandin production connected meloxicam, another clinically available selective COX-2 inhibitor [68], with hematopoiesis and radiation. Meloxicam was found to stimulate hematopoiesis when given in a single dose before irradiation [69,70], or repeatedly after irradiation [69,71]. Details and discussion of these findings can be found in a separate review [2]. It could be deduced from these reports that the hematopoiesis-stimulating effects of the selective COX-2 inhibitors of prostaglandin synthesis retained those of the non-selective ones.

Contrary to the above mentioned aggravated survival of lethally irradiated mice following administration of non-selective COX inhibitors, promising results were obtained when mice exposed to lethal radiation does were given meloxicam, a COX-2 selective NSAID. A significantly increased post-irradiation survival was observed when a single meloxicam dose was administered either shortly (1 h) before or shortly after (1 h) radiation exposure [70,72]. Nevertheless, Jiao et al. [73] published their findings concerning a decreased survival of experimental mice if lethally irradiated animals were administered meloxicam repeatedly, namely seven times during the post-irradiation period. It follows from the observations obtained from survival experiments that dosing and timing of meloxicam in relation to the time of exposure is of crucial importance for obtaining a desirable outcome.

## 8. Summarization and Considerations on Hematological and Radiation-Modulating Effects of Selective COX-2 Inhibitor Meloxicam

Meloxicam is the only selective COX-2 inhibitor investigated in more detail from the point of view of testing its hematopoiesis-stimulating abilities in the situation of the acute radiation syndrome inducing radiation exposure. Therefore, this paragraph deals solely with meloxicam though it could be supposed very roughly that also other selective COX-2 inhibitors could act in relevant situations in a similar way.

Meloxicam’s primary use was especially that in the treatment of rheumatic disease and postoperative pain. It was stated that utilization of meloxicam in these indications was at least as effective as that of the non-selective COX inhibitors (non-selective NSAIDs) but it was accompanied by a more favorable gastrointestinal tolerability [74]. Due to the complexity of the acute radiation syndrome (see Section 3), these findings are highly relevant also from the point of view of the topics of this article. 

As concerns mechanisms, its unquestionable hematopoiesis-stimulating action (see Section 7), the ability of meloxicam to stimulate endogenous production of granulocyte colony-stimulating factor (G-CSF) was revealed and described [70,71,72]. Certainly, also the previously postulated capability of meloxicam to remove the negative feedback control over the production of hematopoietic progenitor cells played by prostaglandins (see Section 6) is of importance in its hematopoiesis-stimulating action.

An intricate issue is that of the timing of meloxicam administration in connection with the attempts to modulate radiation damage in the context of survival experiments. Our group referred about successful improvement of post-irradiation survival when administering meloxicam in a single pre-irradiation dose 1 h before irradiation [70], or in a single post-irradiation dose 1 h after irradiation as a monotherapy [72], as well as in combination with an adenosine A_3_ receptor agonist IB-MECA [75]. We hypothesized that the success of the meloxicam administration nearly immediately after irradiation consisted in its ability to induce G-CSF production. Considered together with the proposal of Hérodin et al. stating that the therapeutic action of hematopoietic cytokines was most beneficial at their action very shortly following the radiation exposure [76,77]. A shift of the single meloxicam injection to the time interval of 24 h after irradiation turned out in nearly equal survival in meloxicam-treated and control mice and, thus, was unsuccessful [70]. Similarly unsuccessful was the extension of the dosing of meloxicam to daily dosing on days 1 to 9 after irradiation [72]. The later result was in accordance with that of Jiao et al. [73] who also tested repeated post-irradiation administration of meloxicam without the desirable effect of the drug on post-irradiation survival. It was hypothesized that the failure of the repeated post-irradiation meloxicam dosing could be due to its vascular [78] or hepatic [79] side effects manifesting at the repeated dosing and a serious post-irradiation stress. At the time interval of about the turn of the first decade of this century it seemed, thus, that the end of the story lay in the recommendation to employ the hematopoiesis-stimulating abilities of selective COX-2 inhibitors in the therapy of acute radiation syndrome in the form of its one-time application shortly after the radiation exposure.

## 9. Considerations Concerning Connecting Pharmacological Interventions with Prostaglandins and Inhibitors of Their Synthesis into One Treatment Scheme

**Figure 2:**
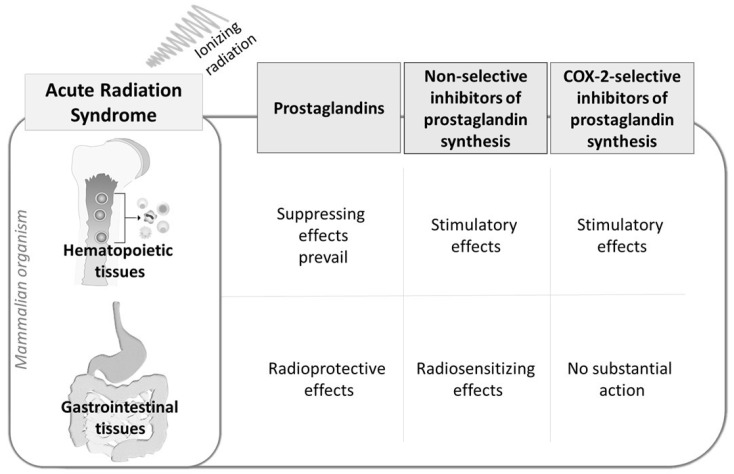
Summarization of the effects of prostaglandins and inhibitors of their synthesis in acute radiation syndrome.

The effects of prostaglandins and inhibitors of their synthesis on acute radiation syndrome are summarized in Figure 2.

A shift in the approach to the topic of this story appeared when dealing simultaneously with hematological and radiation-modulating effects of both prostaglandins and inhibitors of their synthesis. Hoggatt et al. [80] published an article where they presented and discussed the possibilities of how to increase the levels of important hematological parameters and the post-irradiation survival of lethally irradiated mice both by PGE_2_ and by the selective COX-2 inhibitor of prostaglandin synthesis, meloxicam. For PGE_2_, they suggest the treatment regimen of a single PGE_2_ dose at 6 or 24 h after lethal irradiation, for meloxicam that of four daily doses starting 6 or 48 h post-irradiation. As concerns hematological parameters, the authors counted blood platelets and three types of hematopoietic progenitor cells under the above dosing and timing regimens. All the treatment regimens using PGE_2_ or meloxicam showed significantly better survival and status of hematological parameters in comparison with the controls [80]. The PGE_2_ treatment regimen used is, in our opinion, the only one testing post-irradiation treatment with the drug and is in no contradiction to the previous observations on survival of lethally irradiated mice given a PGE_2_ analog before irradiation [37]. However, the enhanced survival following the treatment regimen of repeated post-irradiation doses of meloxicam reported by Hoggatt et al. [80] is in disagreement with the previously observed unchanged or decreased post-irradiation survival after repeated post-irradiation dosing of meloxicam reported by Hofer et al. [72] and Jiao et al. [73]. Consequently, we consider necessary to repeat the investigations on the issue of the profitability of various post-irradiation treatment regimens with meloxicam aimed to define and confirm the best treatment scheme.

Though the structure of the article by Hoggatt et al. [80] invited to make a further step, namely that of combining prostaglandins and inhibitors of their synthesis in the treatment of acute radiation syndrome, no findings have been published interconnecting the administration of prostaglandins and COX inhibitors in a suitable regimen in the course of one experiment. As follows from the previous paragraphs, both prostaglandins and COX inhibitors show hematopoiesis-stimulating and radiation damage-suppressing actions and they achieve their effects by different mechanisms, and following different timing and dosing schedules. Therefore, the idea of their interconnection into one treatment scheme is tempting. It has been repeatedly stated that one of the general approaches to the treatment of acute radiation syndrome aimed at reducing undesirable side effects of the therapy while enhancing the overall therapeutic outcome by combining two (or more) agents [81,82,83]. Combined administration of prostaglandins and inhibitors of their synthesis targeted at stimulation of hematopoiesis and survival enhancement in an irradiated mammalian organism would, thus, represent a promising way in the treatment of acute radiation syndrome and would mean an interesting completing of the story briefly described here.

## 10. Supplementary Note on COX-2-Deficient Mice, Hematopoiesis, and Myelosuppression

Studies examined in the Section 7, Section 8 and Section 9 have dealt with pharmacological inhibition of COX-2 which is acute, potentially not absolute, and may result in partial non-selective co-inhibition of cyclooxygenase-1 [46]. On the other hand, loss of COX-2 activity in COX-2-deficient (COX-2 knock-out, COX-2 KO) mice is life-long, complete, and absolutely selective. First information on behavior of hematopoiesis in COX-2 KO mice appeared in 1989; Lorenz et al. reported on delayed and deteriorated recovery of 5-fluorouracil-induced hematopoietic damage in COX-2 KO mice [84]. In our laboratory we have recently found, using a complex hematological analysis, that in non-treated mice, hematological parameters in COX-2 KO animals are either at the same level compared to wild-type controls or significantly higher in some instances (peripheral blood neutrophils, bone marrow granulocyte/macrophage progenitor cells) [85]. However, in mice with radiation-induced myelosuppression, the overall hematological picture was found to be distinctly worse in the COX-2 KO animals [85]. The latter finding was subsequently supported by the observation of significantly impaired post-irradiation survival of COX-2 KO mice [86]. A hypothesis was formulated that radiation-induced systemic inflammation was beneficial for the post-irradiation hematological recovery and that the inflammation is suppressed by the missing of the inducible COX-2 in COX-2 KO mice [85]. Hematological and radiobiological findings in the COX-2 KO mice with the chronic and absolute COX-2 absence do not have the potential of direct practical use in the clinical medicine. However, they contribute to the understanding of the role of prostaglandins and inhibition of their synthesis in hematopoiesis.

## 11. Conclusions

It can be concluded from the data summarized above that both prostaglandins and inhibitors of their synthesis possess the ability to influence positively acute radiation syndrome in a mammalian organism. The results of recent studies suggest that, at appropriate dosing and timing, administration of prostaglandins and inhibitors of their synthesis could be utilized in one pharmacological treatment regimen with the aim to strengthen the processes of post-irradiation regeneration.

## Figures and Tables

**Table 1:** Approximate values of LD_50/30_ for various mammalian species irradiated with X-rays.

Species	Mouse	Rat	Dog	Monkey (Macaca)	Rabbit	Guinea Pig	Hamster	Pig	Goat	Man
LD_50/30_ (Gy)	6.4	7.1	2.5	6.0	7.5	4.5	6.1	2.5	2.4	3.0 *

* estimated. Modified from Bond et al. [33].
